# Electrode Location in a Microelectrode Recording-Based Model of the Subthalamic Nucleus Can Predict Motor Improvement After Deep Brain Stimulation for Parkinson’s Disease

**DOI:** 10.3390/brainsci9030051

**Published:** 2019-03-01

**Authors:** Rens Verhagen, Lo J. Bour, Vincent J. J. Odekerken, Pepijn van den Munckhof, P. Richard Schuurman, Rob M. A. de Bie

**Affiliations:** 1 Department of Neurology and Clinical Neurophysiology, University of Amsterdam, Amsterdam UMC, Meibergdreef 9, 1105 AZ Amsterdam, The Netherlands; bour@amc.uva.nl (L.J.B.); v.j.odekerken@amc.uva.nl (V.J.J.O.); r.m.debie@amc.uva.nl (R.M.A.d.B.); 2 Department of Neurosurgery, University of Amsterdam, Amsterdam UMC, Meibergdreef 9, 1105 AZ Amsterdam, The Netherlands; p.vandenmunckhof@amc.uva.nl (P.v.d.M.); p.r.schuurman@amc.uva.nl (P.R.S.)

**Keywords:** deep brain stimulation, Parkinson’s disease, subthalamic nucleus

## Abstract

Motor improvement after deep brain stimulation (DBS) in the subthalamic nucleus (STN) may vary substantially between Parkinson’s disease (PD) patients. Research into the relation between improvement and active contact location requires a correction for anatomical variation. We studied the relation between active contact location relative to the neurophysiological STN, estimated by the intraoperative microelectrode recordings (MER-based STN), and contralateral motor improvement after one year. A generic STN shape was transformed to fit onto the stereotactically defined MER sites. The location of 43 electrodes (26 patients), derived from MRI-fused CT images, was expressed relative to this patient-specific MER-based STN. Using regression analyses, the relation between contact location and motor improvement was studied. The regression model that predicts motor improvement based on levodopa effect alone was significantly improved by adding the one-year active contact coordinates (R^2^ change = 0.176, *p* = 0.014). In the combined prediction model (adjusted R^2^ = 0.389, *p* < 0.001), the largest contribution was made by the mediolateral location of the active contact (standardized beta = 0.490, *p* = 0.002). With the MER-based STN as a reference, we were able to find a significant relation between active contact location and motor improvement. MER-based STN modeling can be used to complement imaging-based STN models in the application of DBS.

## 1. Introduction

Deep brain stimulation (DBS) of the subthalamic nucleus (STN) is a widely used and effective surgical treatment for advanced Parkinson’s disease (PD), when treatment with dopaminergic medication is no longer satisfactory [1,2,3]. The main therapeutic effect of STN-DBS lies in the off-medication improvement of the cardinal PD motor symptoms of bradykinesia, tremor, and rigidity [4,5]; an improvement that is on average 50% of the off-medication symptom severity [6]. However, there is a large variation in outcome between patients, both in terms of motor improvement and side effects [2,4,7,8,9]. The postoperative motor improvement is dependent on, amongst others, age and disease duration [10,11,12] as well as the preoperative response to dopaminergic medication [6,12], which makes careful selection of DBS candidates essential. However, the most important factor determining motor improvement reached through DBS is the correct positioning of the active contact in the subthalamic area [13,14,15,16,17,18,19,20,21,22].

The relation between active contact location and motor improvement has been widely studied, but the results of these studies are not conclusive [13]. While some claim that stimulation within the borders of the STN is most effective [15,21,22], others prefer stimulation of the fiber tracts and/or the zone incerta dorsal to the STN [17,19], or specifically the border zone between the dorsal STN and the zona incerta [14,16,18]. The methodologies used to study this relation are variable [13,23]. Determining the active contact location solely in relation to the midcommissural point (MCP) as the anatomical reference ignores a large part of the anatomical variation in STN size and location. This may be the reason that a clear relation between active contact location and motor improvement was not demonstrated with these methods [23,24,25]. The transformation of brain atlases, based on anatomical landmarks, only partially corrects for anatomical variations [13,16,26]. Referring the active contact location to the STN visible in MRI images takes a large part of the anatomical variation into account [20,27,28]. However, this method depends on high quality MRI images, and STN size and location on MRI shows some variation depending on the field strengths and imaging sequences that are used. The dimensions of the MRI-based STN do not always match the dimensions of the neurophysiological STN as measured by intraoperative microelectrode recordings (MER) [29,30,31]. Studies that use MER to define the neurophysiological STN as a reference for the active contact location have been limited in the fact that they only define dorsal and ventral STN borders. Therefore, they are unable to make any statements about the laterality of the active contact location [13,15], while MRI studies have shown that laterality can be an important factor influencing motor improvement [16,20,28].

In this study we defined the location of the active DBS contact after one year relative to an STN model of which the size and location is automatically estimated based on multiple-channel MER measurements (MER-based STN) [31]. This approach enabled us to relate the active contact location not only to the dorsoventral dimensions of the MER-based STN, but also to its anteroposterior and mediolateral dimensions. Then we explored the predictive effect of active contact location relative to this MER-based STN on contralateral motor improvement one year after surgery.

## 2. Materials and Methods

### 2.1. Patient Selection

For this study, we selected a subset of STN implantations (*n* = 45) in patients enrolled in the Netherlands SubThalamic And Pallidal Stimulation (NSTAPS) trial, which compared STN-DBS with Globus Pallidus interna DBS, after receiving ethical approval (ID 07.17.0069, Medical Ethical Committee AMC, 05/17/2006). Additional selection criteria were: (1) patients received STN-DBS in the Academic Medical Center in Amsterdam, (2) three or more MER trajectories measured STN activity during DBS implantation, (3) Unified Parkinson’s Disease Rating Scale (UPDRS) motor scores were available before and one year after surgery, and (4) CT images showing the implanted DBS electrode one year after surgery were available.

### 2.2. Surgical Procedures and Microelectrode Recording (MER)-Based Subthalamic Nucleus (STN) Estimation

DBS surgery was performed using a one-stage unilateral or bilateral stereotactic approach. Standard stereotactic coordinates (12 mm lateral, 2 mm posterior, and 4 mm ventral to MCP) were visually adjusted based on 1.5 Tesla, T2-weighted MRI images. Neurophysiological mapping was performed with a 0.5 mm step size using three (*n* = 20), four (*n* = 19) or five (*n* = 6) tracks of MER to verify STN borders. After MER, macro-electrode test stimulation was performed, and all patients were then implanted with the Medtronic 3389 electrode. Detailed descriptions of the surgery and MER measurements have been published before [32]. All MER measurement sites were scored as either inside or outside the STN by a clinical physicist based on visual analysis of background activity and single/multi-unit spiking activity [33]. A previously developed method was used to automatically estimate patient-specific STN size and the location of all its borders, based on the classifications of multiple-channel MER measurements. This was done in MATLAB^®^ (Mathworks, Inc., Natick, MA, USA) by transposing and scaling an atlas-derived 3D STN shape, within previously validated boundaries [31], to optimally fit the classifications of all MER sites. The resulting MER-based STN was used as the reference for location analysis in this study. The details of this method have been published before [31,34]. This previous publication also showed that a reliable MER-based STN model could be created when STN activity was measured on three or more MER tracks, and that no big differences were found between models created from three, four, or five tracks. The higher resolution in the dorsoventral direction, compared to the anteroposterior and mediolateral directions, is taken into account by the model. If relatively little information is available in the anteroposterior or mediolateral direction, the created model will be closer to the atlas-derived 3D STN with minimal transposing and scaling in these directions. Moreover, a previously published study has shown that the estimated lateral border of the MER-based STN corresponded to the lateral STN border identified on ultra-high field (7.0 T) T2-weighted MRI images [31].

### 2.3. Postoperative Electrode Recognition

Using SureTune^®^ (Medtronic Eindhoven Design Center, The Netherlands), the preoperative T1-MRI images including the stereotactic frame, which were used for preoperative target planning, were co-registered to the CT images showing the DBS electrodes one year after surgery. The software automatically projected a 3D model of the Medtronic 3389 electrode onto the electrode artifact seen on the CT. This projection was manually improved to optimally fit the artifact in three dimensions. After that, the stereotactic electrode location was exported from SureTune^®^ to MATLAB^®^ and combined with the patient-specific MER-based STN in stereotactic space. 

**Figure 1:**
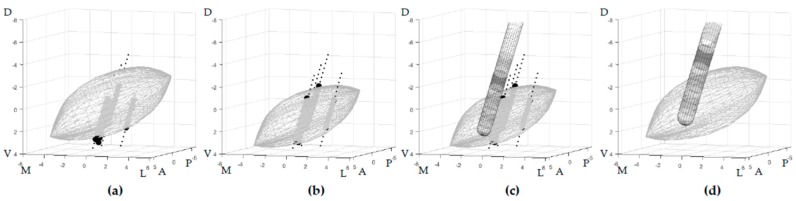
Anterolateral view of the subthalamic nucleus (STN) in an AC–PC-aligned orientation illustrating how the contact locations were expressed in reference to the generalized STN. (**a**) The atlas-derived generic 3D STN shape is placed on top of the patient-specific MER sites (∙ = outside STN, ● = inside STN); (**b**) the STN is transformed to optimally fit the classifications of all MER sites; (**c**) the DBS electrode and its contacts, recognized in MRI-fused CT images, are combined with the MER-based STN using the patient-specific stereotactic frame (active contact after one year in darker gray); (**d**) both the MER-based STN and the recognized electrode are inversely transformed together. All the electrode contacts are now expressed in reference to the generalized STN in an AC–PC-aligned orientation. (A = anterior, D = dorsal, L = lateral, M = medial, P = posterior, and V = ventral, locations displayed on the axes are in mm).

In this study, we calculated the position of the stimulated contact one year after surgery relative to each patient’s specific MER-based STN. In order to perform a group analysis, every patient-specific STN was transformed, together with the electrode location, back to the initial STN shape from which it was created. This created a generic STN shape in an anterior commissure—posterior commissure (AC–PC) -aligned orientation in reference to which all the locations were expressed, hereafter referred to as the generalized STN. By doing this, we compensated for the anatomical variation in STN size and location. The back-transformation was done using the inverse of the transposition and scaling applied to initially produce the patient-specific MER-based STN. The electrode was transformed with the same inverse transformations so that the contact locations of all patients were expressed in reference to the same generalized STN, while the position of the contact locations relative to this STN remained to be based on the patient-specific MER measurements (Figure 1). For right-hemisphere implantation, all procedures were mirrored to enable a group analysis.

The contact used for stimulation was retrieved from the stimulator settings after a neurologist optimized them over the course of one year. When bipolar stimulation was used (*n* = 3), the negative pole was determined as the active contact. In double monopolar stimulation (*n* = 2), the center between the two active contacts was used as the active contact location. To verify our results, the analysis was also performed without these five electrodes.

### 2.4. Motor Improvement

In the NSTAPS trial, PD motor symptoms were assessed using the UPDRS part III after optimization of the stimulation parameters over the course of one year. Both patients and clinical raters were blinded for active contact location [4]. For this study on the contralateral motor improvement, only the purely one-sided UPDRS motor score sub-items (UPDRS-III items 20–26) were used [35]. Because off-medication motor improvement is typical for effective STN-DBS, the motor improvement was studied by the off-medication stimulation effect, which is defined by the change in off-medication hemibody UPDRS motor score after DBS as a percentage of the baseline off-medication hemibody UPDRS motor score determined preoperatively:

(1) $$\mathrm{Stimulation}\;\mathrm{effect}=\frac{{\mathrm{hemibody}\;\mathrm{UPDRS}}_{\mathrm{med}\_\mathrm{off}\;}\left(\mathrm{baseline}\right){-\mathrm{hemibody}\;\mathrm{UPDRS}}_{\mathrm{med}\_\mathrm{off}|\mathrm{stim}\_\mathrm{on}\;}\left(1\;\mathrm{year}\right)}{{\mathrm{hemibody}\;\mathrm{UPDRS}}_{\mathrm{med}\_\mathrm{off}}\;\left(\mathrm{baseline}\right)}$$

### 2.5. Statistical Analysis

The coordinates of the active contacts relative to the center of the generalized STN were statistically analyzed in an AC–PC-aligned three-dimensional coordinate system with the *x*-axis from medial to lateral, *y*-axis from posterior to anterior, and the *z*-axis from dorsal to ventral.

To analyze the predictive power of active contact location, multiple linear regression was performed using SPSS version 23 (SPSS Inc., Chicago, IL, USA), with the off-medication stimulation effect as the dependent variable and the three coordinates (*x*, *y,* and *z*) of the active contact locations relative to the center of the generalized STN as independent variables. Because the preoperative response to dopaminergic medication is a known predictor for the off-medication stimulation effect, the levodopa window (hemibody UPDRS_med_off_ (baseline) − hemibody UPDRS_med_on_ (baseline)) was also included as an independent variable. Hierarchical multiple regression was performed to study the added predictive value of the three active contact coordinates after controlling for the influence of the levodopa window.

## 3. Results

Of the 45 implanted electrodes from the NSTAPS trial that matched our inclusion criteria, one was excluded because the MER classifications of different trajectories were conflicting, and we were unable to produce a reliable MER-based STN estimation. Furthermore, regression analysis revealed one negative outlier in the dependent variable stimulation effect (−133%). Because of the profound impact that outliers can have on linear regression fits, possibly causing overestimation or underestimation of the relation, this observation was excluded in all further regression analyses. 

The relation between active contact location and contralateral off-medication stimulation effect was studied in 26 patients (43 electrodes). Clinical characteristics are summarized in Table 1. The three-dimensional locations of the active contacts one year after surgery, relative to the center of the generalized STN, are summarized in Table 2.

**Figure 2:**
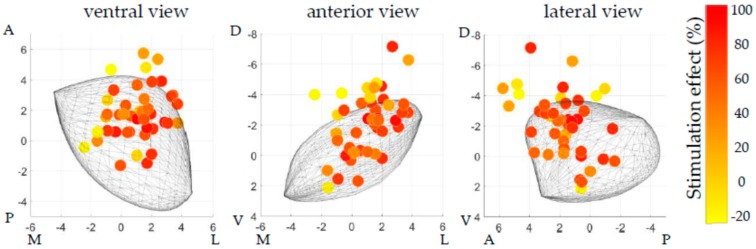
Distribution of the active contact locations after one year in a ventral, anterior, and lateral view. The gray shape represents the generalized STN, identical for all patients. Locations are color-coded corresponding to their contralateral off-medication stimulation effect. Overall, the distribution of the locations is centered on the anterior part of the dorsolateral STN. The relation with stimulation effect is most striking in the anterior view, where the locations at the lateral and ventral part of the distribution often correspond to good stimulation effect (red), while locations at the medial and dorsal part of the distribution often corresponding to poor stimulation effect (yellow). (A = anterior, D = dorsal, L = lateral, M = medial, P = posterior, and V = ventral, locations displayed on the axes are in mm).

**Figure 3:**
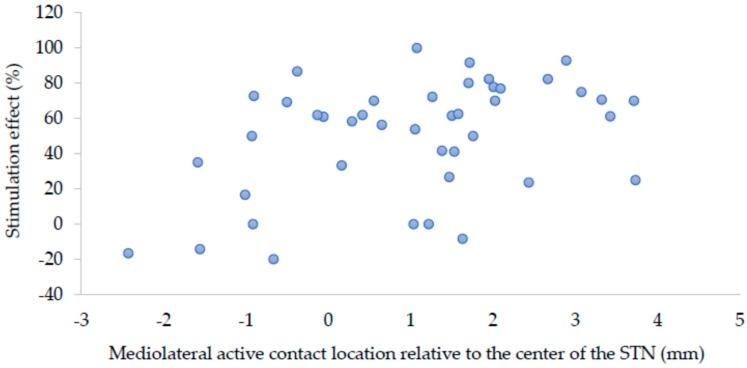
Scatter plot of the relation between mediolateral active contact location (*x*-coordinate) relative to the center of the STN, and stimulation effect.

Hierarchical regression revealed that adding the three coordinates of the active contact to the known predictor levodopa window resulted in a significant improvement of the prediction of off-medication stimulation effect (R^2^ change = 0.176, *p* = 0.014). The combined model, with the levodopa window and the three active contact coordinates (*x*, *y,* and *z*) as the independent variables, was a significant predictor of off-medication stimulation effect (adjusted R^2^ = 0.389, *p* < 0.001). In this model, the mediolateral location (*x*-coordinate) of the active contact had the largest unique contribution to the prediction (standardized beta = 0.490, *p* = 0.002), followed by the levodopa window (standardized beta = 0.328, *p* = 0.022). The contributions of the anteroposterior (*y*-coordinate) and dorsoventral location (*z*-coordinate) of the active contact were not significant. Figure 2 displays the distribution of the active contact locations after one year relative to the generalized STN, which were used for the regression analysis. Figure 3 shows a scatter plot of the relation between mediolateral location (*x*-coordinate) relative to the center of the STN and stimulation effect. 

The same analysis without exclusion of the outlier in stimulation effect resulted in the same significant relations, with even slightly higher values for R^2^ change and adjusted R^2^. Thereby, exclusion of this single observation was justifiable on theoretical grounds, but it had no effect on the relations found in this study.

The same analysis with exclusion of the bipolar (*n* = 3) and double monopolar (*n* = 2) stimulating electrodes led to similar main results. Namely, a significant improvement in the prediction was found by adding the coordinates (R^2^ change = 0.186, *p* = 0.041), and this resulted in a significant combined prediction model (adjusted R^2^ = 0.254, *p* < 0.008) in which the mediolateral location has the largest unique contribution (standardized beta = 0.388, *p* = 0.044). Furthermore, in this subgroup analysis, the anteroposterior location (*y*-coordinate) had a significant unique contribution to the prediction (standardized beta = −0.341, *p* = 0.048), whereas the unique contribution of the levodopa window was no longer significant.

Using the active contact locations corresponding to profound off-medication stimulation effects (≥75%, *n* = 10), a theoretical hotspot for DBS could be estimated at 1.9 mm lateral, 0.9 mm anterior, and 2.6 mm dorsal to the center of the generalized MER-based STN. When the same active contacts with profound effect were related to MCP, this resulted in a theoretical hotspot at 12.6 mm lateral, 2.0 mm posterior, and 2.8 mm ventral to MCP.

## 4. Discussion

Using the neurophysiological borders based on MER to create individual STN models as a reference for active contact location, we found a significant relation between active contact location and change in contralateral off-medication motor score with DBS compared to baseline. This is in contrast to earlier studies that have used active contact location relative to MCP and have reported no relation with motor improvement. Failure to find a relation in these earlier studies is likely the result of anatomical variation in STN location between patients [23,24,25,26]. This indicates that the MER-based correction of STN-modelling can result in a valid reference for active contact location. Stimulation within the lateral part of the MER-based STN resulted in more substantial motor improvement compared to stimulation more medial within the MER-based STN, confirming similar imaging-based analyses.

Our regression analysis showed that the coordinates of the active contact, relative to the MER-based STN, explained an additional 17.6% of the variance in off-medication stimulation effect after controlling for the influence of a levodopa window. Together, they explain 38.9% of the variance in off-medication stimulation effect. Of these three coordinates, the laterality (*x*-coordinate) has the greatest unique contribution to the regression model, greater than that of the levodopa window. This can be explained by the anatomical location of the sensorimotor area in the lateral part of the STN, while the associative and limbic areas of the STN are more medial [36,37]. The positive influence on stimulation effect of a more lateral active contact corresponds with findings that use the MRI-based STN as a reference [20,28,38]. 

In the analysis where bipolar and double monopolar stimulating electrodes were excluded, the findings described above were confirmed. Furthermore, the anteroposterior location of the active contact was found to also have a significant, unique contribution to the regression model, where a more posterior active contact was related to more stimulation effects. This is in line with research showing that sensorimotor projections of the STN are more posteriorly located [36,37]. In this subgroup analysis, the levodopa window had a non-significant contribution to the regression model. This might be explained by the decreased number of analyzed electrodes compared to the full analysis. Furthermore, for both of the analyses, it should be noted that the influence of the levodopa window might be underestimated because patients were selected for DBS based on, amongst others, a large levodopa window. The location results remain valid for this group, but when considering all PD patients eligible for DBS surgery, the levodopa window is likely to remain the most important predictor for postoperative motor improvement.

The advantage of using the MER-based STN as a reference is that it derives from real-time intraoperative physiological data from the patient, confirming the location of the STN rather than relying on an indirect pre-operative dataset that can be subject to distortion (as MRI can be). Furthermore, the DBS electrode location related to the MER-based STN can easily be assessed intraoperatively, even when the MER-based STN does not correspond with the MRI-based STN. In our center, the symptomatic bleeding rate with this MER driven approach was 0.8%. 

To compare implantation in our center with other centers, we also calculated the active contact locations referenced to MCP. The mean location in our patients (12.0 mm lateral; 1.0 mm posterior; 3.1 mm ventral) corresponded reasonably well with the average of eight earlier studies presented in a review by Caire et al. (12.0 mm lateral; 1.5 mm posterior; 1.9 mm ventral) [13]. However, active contact locations in our group were slightly more ventral, which likely results from the fact that the targeting in our center was done with the intention to stimulate inside the STN, compared to some other centers where stimulation dorsal to the STN was intended. Thus, while our theoretical hotspot was dorsal to the center of the STN, but within its boundaries, other centers target even more dorsal (and often more posterior) to stimulate fiber tracts outside the STN boundaries. Therefore, it should be noted that the relation between active contact location and motor improvement found here is particularly valid for STN-DBS surgery using similar targeting and surgical procedures as in our center. Most importantly, these procedures include the intended stimulation within the boundaries of the STN and intraoperative refinement of STN borders by MER. Other groups might target different structures, for example more posterior or dorsal to the STN, or use different forms of intraoperative visualization of the STN. Therefore, generalization of these results to all STN-DBS should be considered with caution. 

In this study, in 26 patients, the 43 electrodes and its effects on motor improvement were treated as independent. This assumes that the effect of one-sided stimulation on the purely one-sided contralateral UPDRS motor score sub-items is independent of the stimulation of the other STN in the same patient. Any dependency between these effects may have influenced the statistical significance.

Furthermore, to study solely the stimulation effect of STN-DBS, the off-medication on-stimulation motor scores after one year would have to be compared to the off-medication off-stimulation motor scores after one year. Since the latter were not available in this cohort, the off-medication scores at baseline were used. Therefore, possible effects of disease progression, microlesions, or long-term medication effects are also included in the outcome parameter called stimulation effect in this study, which may have led to both overestimation and underestimation of the pure effect of stimulation alone. 

## 5. Conclusions

Our study showed that, using the neurophysiological boundaries of the STN based on MER, it is possible to create a model of the STN as a reference for active DBS contact location based on real-time, intraoperatively acquired neurophysiological data from the patient. There is a clear correlation between these locations and motor improvement after STN-DBS, whereby more lateral stimulation within the boundaries of the MER-based STN predicts more motor improvement after one year. This result is in contrast to earlier studies that have used active contact location relative to MCP and have reported no relation with motor improvement.

The neurophysiological generalized model of the STN can be used to complement imaging studies in the search for better clinical outcomes of DBS. Other neurophysiological modalities, like local field potential recordings, can be added to the model, and can become important in future stimulation paradigms of steerable or adaptive deep brain stimulation.

## Figures and Tables

**Table 1:** Clinical characteristics of the patients (top) and the contralateral hemibody scores per electrode (bottom).

**Patient Characteristics (*n* = 26)**	
Male/Female	20/6
Age—years (mean ± SD)	62.7 ± 7.1
Disease Duration—years (mean ± SD)	12.6 ± 6.6
**Contralateral Hemibody Scores Per Electrode (*n* = 43)**	
Levodopa Window (mean ± SD)	10.5 ± 5.5
Baseline UPDRS_med_off_ (score range = 0–36) (mean ± SD)	15.0 ± 5.6
One-year UPDRS_med_off_ (score range = 0–36] (mean ± SD)	6.6 ± 4.2
Off-medication stimulation effect—% (mean ± SD)	50.3 ± 32.5

UPDRS: Unified Parkinson’s Disease Rating Scale.

**Table 2:** The three-dimensional active contact locations after one year.

Direction	Location to Center STN mm (Mean ± SD) ^1^
Mediolateral (*x*)	1.0 ± 1.5
Anteroposterior (*y*)	1.7 ± 1.7
Dorsoventral (*z*)	−2.2 ± 2.1

^1^ Distances are referenced to the center of the generalized STN. The *x*-coordinate is positive towards lateral, *y* is positive towards anterior, and *z* is positive towards ventral.

## References

[1] Benabid A.L., Chabardes S., Mitrofanis J., Pollak P. (2009). Deep brain stimulation of the subthalamic nucleus for the treatment of Parkinson’s disease. Lancet Neurol..

[2] Deuschl G., Schade-Brittinger C., Krack P., Volkmann J., Schäfer H., Bötzel K., Daniels C., Deutschländer A., Dillmann U., Eisner W. (2006). A Randomized Trial of Deep-Brain Stimulation for Parkinson’s Disease. N. Engl. J. Med..

[3] Limousin P., Krack P., Pollak P., Benazzouz A., Ardouin C., Hoffmann D., Benabid A.-L. (1998). Electrical stimulation of the subthalamic nucleus in advanced Parkinson’s disease. N. Engl. J. Med..

[4] Odekerken V.J.J., van Laar T., Staal M.J., Mosch A., Hoffmann C.F.E., Nijssen P.C.G., Beute G.N., van Vugt J.P.P., Lenders M.W.P.M., Contarino M.F. (2013). Subthalamic nucleus versus globus pallidus bilateral deep brain stimulation for advanced Parkinson’s disease (NSTAPS study): A randomised controlled trial. Lancet Neurol..

[5] Follett K.A., Weaver F.M., Stern M., Hur K., Harris C.L., Luo P., Marks W.J., Rothlind J., Sagher O., Moy C. (2010). Pallidal versus Subthalamic Deep-Brain Stimulation for Parkinson’s Disease. N. Engl. J. Med..

[6] Kleiner-Fisman G., Herzog J., Fisman D.N., Tamma F., Lyons K.E., Pahwa R., Lang A.E., Deuschl G. (2006). Subthalamic nucleus deep brain stimulation: Summary and meta-analysis of outcomes. Mov. Disord..

[7] Odekerken V.J.J., Boel J.A., Geurtsen G.J., Schmand B.A., Dekker I.P., de Haan R.J., Schuurman P.R., de Bie R.M.A. (2015). Neuropsychological outcome after deep brain stimulation for Parkinson disease. Neurology.

[8] Williams A., Gill S., Varma T., Jenkinson C., Quinn N., Mitchell R., Scott R., Ives N., Rick C., Daniels J. (2010). Deep brain stimulation plus best medical therapy versus best medical therapy alone for advanced Parkinson’s disease (PD SURG trial): A randomised, open-label trial. Lancet Neurol..

[9] Weaver F.M., Follett K., Stern M., Hur K., Harris C., Marks W.J., Rothlind J., Sagher O., Reda D., Moy C.S. (2009). Bilateral deep brain stimulation vs best medical therapy for patients with advanced parkinson disease: A randomized controlled trial. JAMA.

[10] Aygun D., Kocabicak E., Yildiz M.O., Temel Y. (2016). Effect of age and disease duration on the levodopa response in patients with advanced Parkinson’s disease for deep brain stimulation of the subthalamic nucleus. Front. Neurol..

[11] Parent B., Awan N., Berman S.B., Suski V., Moore R., Crammond D., Kondziolka D. (2011). The relevance of age and disease duration for intervention with subthalamic nucleus deep brain stimulation surgery in Parkinson disease. J. Neurosurg..

[12] Bronstein J.M., Tagliati M., Alterman R.L., Lozano A.M., Volkmann J., Stefani A., Horak F.B., Okun M.S., Foote K.D., Krack P. (2011). Deep brain stimulation for Parkinson disease an expert consensus and review of key issues. Arch. Neurol..

[13] Caire F., Ranoux D., Guehl D., Burbaud P., Cuny E. (2013). A systematic review of studies on anatomical position of electrode contacts used for chronic subthalamic stimulation in Parkinson’s disease. Acta Neurochir..

[14] Hamel W., Fietzek U., Morsnowski A., Schrader B., Herzog J., Weinert D., Pfister G., Müller D., Volkmann J., Deuschl G. (2003). Deep brain stimulation of the subthalamic nucleus in Parkinson’s disease: Evaluation of active electrode contacts. J. Neurol. Neurosurg. Psychiatry.

[15] Herzog J., Fietzek U., Hamel W., Morsnowski A., Steigerwald F., Schrader B., Weinert D., Pfister G., Müller D., Mehdorn H.M. (2004). Most effective stimulation site in subthalamic deep brain stimulation for Parkinson’s disease. Mov. Disord..

[16] Maks C.B., Butson C.R., Walter B.L., Vitek J.L., McIntyre C.C. (2009). Deep brain stimulation activation volumes and their association with neurophysiological mapping and therapeutic outcomes. J. Neurol. Neurosurg. Psychiatry.

[17] Plaha P., Ben-Shlomo Y., Patel N.K., Gill S.S. (2006). Stimulation of the caudal zona incerta is superior to stimulation of the subthalamic nucleus in improving contralateral parkinsonism. Brain.

[18] Vergani F., Landi A., Antonini A., Parolin M., Cilia R., Grimaldi M., Ferrarese C., Gaini S.M., Sganzerla E.P. (2007). Anatomical identification of active contacts in subthalamic deep brain stimulation. Surg. Neurol..

[19] Voges J., Volkmann J., Allert N., Lehrke R., Koulousakis A., Freund H.-J., Sturm V. (2002). Bilateral high-frequency stimulation in the subthalamic nucleus for the treatment of Parkinson disease: correlation of therapeutic effect with anatomical electrode position. J. Neurosurg..

[20] Wodarg F., Herzog J., Reese R., Falk D., Pinsker M.O., Steigerwald F., Jansen O., Deuschl G., Mehdorn H.M., Volkmann J. (2012). Stimulation site within the MRI-defined STN predicts postoperative motor outcome. Mov. Disord..

[21] Johnsen E.L., Sunde N., Mogensen P.H., Østergaard K. (2010). MRI verified STN stimulation site—Gait improvement and clinical outcome. Eur. J. Neurol..

[22] Zonenshayn M., Sterio D., Kelly P.J., Rezai A.R., Beric A. (2004). Location of the active contact within the subthalamic nucleus (STN) in the treatment of idiopathic Parkinson’s disease. Surg. Neurol..

[23] Nestor K.A., Jones J.D., Butson C.R., Morishita T., Jacobson C.E., Peace D.A., Chen D., Foote K.D., Okun M.S. (2014). Coordinate-based lead location does not predict parkinson’s disease deep brain stimulation outcome. PLoS One.

[24] McClelland S., Ford B., Senatus P.B., Winfield L.M., Du Y.E., Pullman S.L., Yu Q., Frucht S.J., McKhann G.M., Goodman R.R. (2005). Subthalamic stimulation for Parkinson disease: Determination of electrode location necessary for clinical efficacy. Neurosurg. Focus.

[25] Sun H.P., Jung H.H., Lee J.Y., Kim C., Beom S.J., Dong G.K. (2008). Electrode position determined by fused images of preoperative and postoperative magnetic resonance imaging and surgical outcome after subthalamic nucleus deep brain stimulation. Neurosurgery.

[26] Kasasbeh A., Abulseoud O.A., Matsumoto J.Y., Stead S.M., Goerss S.J., Klassen B.T., Huston J., Min H.K., Lee K.H., Frye M.A. (2013). Lack of differential motor outcome with subthalamic nucleus region stimulation in Parkinson’s disease. J. Clin. Neurosci..

[27] Bot M., Schuurman P.R., Odekerken V.J.J., Verhagen R., Contarino F.M., De Bie R.M.A., van den Munckhof P. (2018). Deep brain stimulation for Parkinson’s disease: Defining the optimal location within the subthalamic nucleus. J. Neurol. Neurosurg. Psychiatry.

[28] Garcia-Garcia D., Guridi J., Toledo J.B., Alegre M., Obeso J.A., Rodriguez-Oroz M.C. (2016). Stimulation sites in the subthalamic nucleus and clinical improvement in Parkinson’s disease: A new approach for active contact localization. J. Neurosurg..

[29] Hamani C., Richter E.O., Andrade-Souza Y., Hutchison W., Saint-Cyr J.A., Lozano A.M. (2005). Correspondence of microelectrode mapping with magnetic resonance imaging for subthalamic nucleus procedures. Surg. Neurol..

[30] Polanski W.H., Martin K.D., Engellandt K., von Kummer R., Klingelhoefer L., Fauser M., Storch A., Schackert G., Sobottka S.B. (2015). Accuracy of subthalamic nucleus targeting by T2, FLAIR and SWI-3-Tesla MRI confirmed by microelectrode recordings. Acta Neurochir..

[31] Verhagen R., Schuurman P.R., Van Den Munckhof P., Contarino M.F., De Bie R.M.A., Bour L.J. (2016). Comparative study of microelectrode recording-based STN location and MRI-based STN location in low to ultra-high field (7.0 T) T2-weighted MRI images. J. Neural Eng..

[32] Contarino M.F., Bour L.J., Bot M., Van Den Munckhof P., Speelman J.D., Schuurman P.R., De Bie R.M. (2012). Tremor-specific neuronal oscillation pattern in dorsal subthalamic nucleus of parkinsonian patients. Brain Stimul..

[33] Cagnan H., Dolan K., He X., Contarino M.F., Schuurman R., Van Den Munckhof P., Wadman W.J., Bour L., Martens H.C. (2011). Automatic subthalamic nucleus detection from microelectrode recordings based on noise level and neuronal activity. J. Neural Eng..

[34] Lourens M.A.J., Meijer H.G.E., Contarino M.F., van den Munckhof P., Schuurman P.R., van Gils S.A., Bour L.J. (2013). Functional neuronal activity and connectivity within the subthalamic nucleus in Parkinson’s disease. Clin. Neurophysiol..

[35] Little S., Pogosyan A., Kuhn A.A., Brown P. (2012). Beta band stability over time correlates with Parkinsonian rigidity and bradykinesia. Exp. Neurol..

[36] Hamani C., Saint-Cyr J.A., Fraser J., Kaplitt M., Lozano A.M. (2004). The subthalamic nucleus in the context of movement disorders. Brain.

[37] Parent A., Hazrati L.N. (1995). Functional anatomy of the basal ganglia. II. The place of subthalamic nucleus and external pallidium in basal ganglia circuitry. Brain Res. Rev..

[38] Aviles-Olmos I., Kefalopoulou Z., Tripoliti E., Candelario J., Akram H., Martinez-Torres I., Jahanshahi M., Foltynie T., Hariz M., Zrinzo L. (2014). Long-term outcome of subthalamic nucleus deep brain stimulation for Parkinson’s disease using an MRI-guided and MRI-verified approach. J. Neurol. Neurosurg. Psychiatry.

